# Effectiveness of Four Different Interventions Against *Schistosoma haematobium* in a Seasonal Transmission Setting of Côte d’Ivoire: A Cluster Randomized Trial

**DOI:** 10.1093/cid/ciab787

**Published:** 2021-09-14

**Authors:** Mamadou Ouattara, Fidèle K Bassa, Nana R Diakité, Jan Hattendorf, Jean T Coulibaly, Patrick K Yao, Yves-Nathan T Tian-Bi, Cyrille K Konan, Rufin K Assaré, Naférima Koné, Négnorogo Guindo-Coulibaly, Jürg Utzinger, Eliézer K N’Goran

**Affiliations:** Unité de Formation et de Recherche Biosciences, Université Félix Houphouët-Boigny, Abidjan, Côte d’Ivoire; Centre Suisse de Recherches Scientifiques en Côte d’Ivoire, Abidjan, Côte d’Ivoire; Unité de Formation et de Recherche Biosciences, Université Félix Houphouët-Boigny, Abidjan, Côte d’Ivoire; Centre Suisse de Recherches Scientifiques en Côte d’Ivoire, Abidjan, Côte d’Ivoire; Unité de Formation et de Recherche Biosciences, Université Félix Houphouët-Boigny, Abidjan, Côte d’Ivoire; Centre Suisse de Recherches Scientifiques en Côte d’Ivoire, Abidjan, Côte d’Ivoire; Swiss Tropical and Public Health Institute, Basel, Switzerland; University of Basel, Basel, Switzerland; Unité de Formation et de Recherche Biosciences, Université Félix Houphouët-Boigny, Abidjan, Côte d’Ivoire; Centre Suisse de Recherches Scientifiques en Côte d’Ivoire, Abidjan, Côte d’Ivoire; Swiss Tropical and Public Health Institute, Basel, Switzerland; University of Basel, Basel, Switzerland; Unité de Formation et de Recherche Biosciences, Université Félix Houphouët-Boigny, Abidjan, Côte d’Ivoire; Unité de Formation et de Recherche Biosciences, Université Félix Houphouët-Boigny, Abidjan, Côte d’Ivoire; Centre Suisse de Recherches Scientifiques en Côte d’Ivoire, Abidjan, Côte d’Ivoire; Unité de Formation et de Recherche Biosciences, Université Félix Houphouët-Boigny, Abidjan, Côte d’Ivoire; Centre Suisse de Recherches Scientifiques en Côte d’Ivoire, Abidjan, Côte d’Ivoire; Unité de Formation et de Recherche Biosciences, Université Félix Houphouët-Boigny, Abidjan, Côte d’Ivoire; Centre Suisse de Recherches Scientifiques en Côte d’Ivoire, Abidjan, Côte d’Ivoire; Unité de Formation et de Recherche Biosciences, Université Félix Houphouët-Boigny, Abidjan, Côte d’Ivoire; Unité de Formation et de Recherche Biosciences, Université Félix Houphouët-Boigny, Abidjan, Côte d’Ivoire; Swiss Tropical and Public Health Institute, Basel, Switzerland; University of Basel, Basel, Switzerland; Unité de Formation et de Recherche Biosciences, Université Félix Houphouët-Boigny, Abidjan, Côte d’Ivoire; Centre Suisse de Recherches Scientifiques en Côte d’Ivoire, Abidjan, Côte d’Ivoire

**Keywords:** schistosomiasis, *Schistosoma haematobium*, seasonal transmission, interruption of transmission, Côte d’Ivoire

## Abstract

**Background:**

Annual mass drug administration (MDA) using praziquantel is the cornerstone of schistosomiasis morbidity control but is not sufficient to interrupt transmission. We implemented a cluster-randomized trial to compare the effectiveness of 4 different intervention packages to interrupt transmission of *Schistosoma haematobium* in a seasonal transmission setting of Côte d’Ivoire.

**Methods:**

Sixty-four localities with a *S. haematobium* prevalence in school children aged 13–14 years above 4% were randomly assigned to 1 of 4 intervention arms over a 3-year period: (1) the current standard strategy consisting of annual MDA before peak of transmission, (2) annual MDA after peak of transmission, (3) biannual MDA, and (4) standard MDA combined with snail control. The primary outcome was prevalence and intensity of *S. haematobium* infection in children aged 9–12 years 1 year after the final intervention, using urine filtration performed by experienced microscopists.

**Results:**

By study end, we observed the lowest *S. haematobium* prevalence in the biannual MDA, compared to the standard treatment arm (0.6% vs 7.5%; odds ratio [OR] = 0.07, 95% confidence interval [CI] = .02 to .24). The prevalence in arms 2 and 4 was about 3.5%, which was not statistically significantly different from the standard strategy (both ORs 0.4, 95% CI = .1 to ~1.8). New cases of infection were still observed in all arms at study end.

**Conclusions:**

Biannual MDA was the only regimen that outperformed the standard treatment. All strategies resulted in decreased prevalence of infection; however, none of them was able to interrupt transmission of *S. haematobium* within a 3-year period.

**Clinical Trials Registration:**

ISRCTN10926858.

## Teaser

Four different intervention packages substantially reduced the prevalence and intensity of *Schistosoma haematobium* infection in a seasonal transmission area. However, none achieved interruption of transmission. Snail control failed to boost the impact of preventive chemotherapy in this 3-year cluster-randomized trial.

Schistosomiasis is a neglected tropical disease causing a considerable public health burden [1, 2]. It primarily occurs in tropical and subtropical areas of Africa, where, in 2002, an estimated 436 and 393 million people were affected by *Schistosoma haematobium* and *Schistosoma mansoni*, respectively [3]. Preventive chemotherapy with praziquantel is the mainstay of the global control strategy [4]. With partners supporting a roadmap put forth by the World Health Organization (WHO) [5], considerable progress has been made over the past 15 years [6]. New goals were set by WHO in line with World Health Assembly resolution 65.21 [7]; namely, (i) schistosomiasis elimination as a public health problem (prevalence of heavy infections <1%) and (ii) interruption of transmission (zero new cases of infection) in selected areas by 2025 [6]. Moving toward elimination might require a combination of preventive chemotherapy with other measures, such as snail control, water, sanitation, and hygiene; information, education, and communication [8–10].

The Schistosomiasis Consortium for Operational Research and Evaluation (SCORE) supported 2 large cluster-randomized trials in Côte d’Ivoire. A first trial aimed at sustaining the control of *S. mansoni* with different mass drug administration (MDA) schemes [11–13]. The primary aim of the trial reported here was to assess the effectiveness of different schedules of MDA with or without snail control in interrupting *S. haematobium* transmission in settings characterized by seasonal transmission. The difference in prevalence and intensity of *S. haematobium* infection between the study arms was evaluated after 3 years of intervention in children aged 9–12 years, as this age group is considered at high risk of schistosomiasis. Elimination as public health problem and the potential added value of snail control were explored as secondary objectives. In addition, effects of interventions were assessed on first-grade children (aged 5–8 years) and adults (aged 20–55 years).

## METHODS

### Ethics Statement

Approval for the study was obtained from the ethics committees in Côte d’Ivoire (Comité National d’Éthique et de la Recherche; reference no. 113/MSLS/CNER-dkn, by 22 January 2015) and Switzerland (Ethikkommission Nordwest- und Zentralschweiz; reference no. UBE-15/34, by 15 April 2015). For the use of the molluscicide niclosamide, the study obtained approval of the Direction Générale des Productions et de la Sécurité Alimentaire (reference no. 0163/MINAGRI/DGPSA/DPVCQ, by 27 January 2015).

The trial was registered at the International Standard Randomized Controlled Trial Number (ISRCTN) by 21 December 2016 (reference no. ISRCTN10926858). Written informed consent was obtained from adults and parents/guardians of children aged 5–14 years. Participation was voluntary, and data were kept confidential by using participants’ individual codes instead of the names.

### Study Area and Population

Details on the study populations and the eligibility criteria have been described elsewhere [14, 15]. In brief, the eligibility criteria of the study villages were: (i) location in a *S. haematobium* seasonal transmission area; (ii) presence of a primary school attended by at least 100 pupils aged 9–12 years; and (iii) *S. haematobium* prevalence of at least 4% among 50 screened children aged 13–14 years. The northern and central parts of Côte d’Ivoire were eligible, considering the marked seasonality consisting in a rainy season occurring from April to October and a dry season from November to March, an annual average temperature above 25°C and an average annual precipitation that ranges from 1115 to 1260 mm. The main activity of people in this part of Côte d’Ivoire is subsistence farming. Prior studies carried out in northern and central Côte d’Ivoire revealed low or moderate endemicity of *S. haematobium* [16–18].

**Figure 1.**
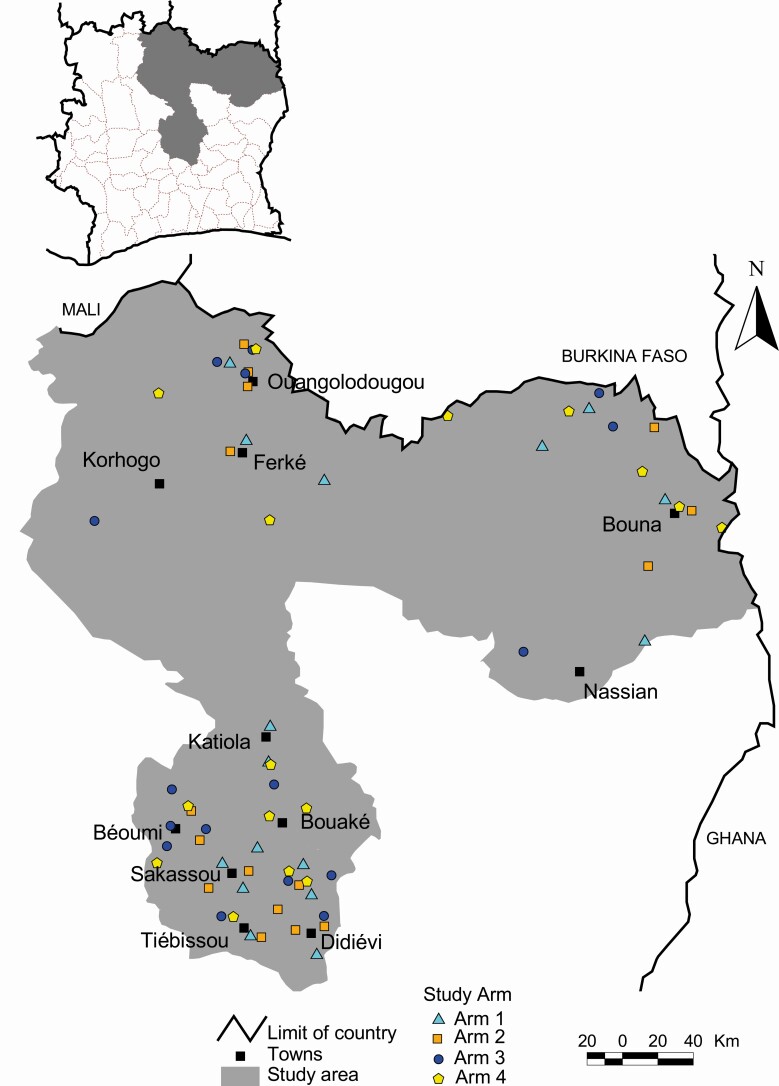
Map of the study area showing the 64 study villages in northern and central parts of Côte d’Ivoire by intervention arms. Arm 1: Annual MDA with praziquantel before the peak schistosomiasis transmission season. Arm 2: Annual MDA with praziquantel after the peak schistosomiasis transmission season. Arm 3: MDA biannual treatment. Arm 4: Annual MDA with praziquantel before the peak schistosomiasis transmission season plus snails control with niclosamide. Abbreviation: MDA, mass drug administration.

Sixty-four villages of the administrative regions of the northern (Tchologo, Poro, Bounkani, and Hambol) and the central (Gbêkê and Bélier) parts of Côte d’Ivoire were selected. Although the study protocol called for enrollment of 60 villages, 64 villages met enrollment criteria and were included because of promises made during the consenting for the screening survey. The inclusion of the 4 additional villages was approved by funders (SCORE). Thus, there were 16 villages (instead of 15 according to the study protocol) per study arm (Figure 1).

### Study Design and Interventions

The study was a 3-year cluster-randomized trial with 4 intervention arms: (1) villages received annual MDA with praziquantel before the peak of schistosomiasis transmission season (this can be considered as the recommended standard of care in this setting and is therefore used as reference arm); (2) annual MDA with praziquantel after the peak of schistosomiasis transmission season; (3) biannual treatments before and after the peak of schistosomiasis transmission season; and (4) annual MDA with praziquantel before the peak of schistosomiasis transmission season, coupled with snail control using niclosamide.

A few days before each MDA, a parasitologic survey was conducted in the study villages among children aged 5–12 years and adults aged 20–55 years, except for the second MDA in arm 3. The final parasitologic data collection was pursued in each village, 1 year after the last MDA by each study arm.

Difference in *S. haematobium* prevalence and intensity of infection between study arms was assessed in the 3 study groups at the final survey with the primary objective focused on 9- to 12-year-old children.

### Parasitologic Survey

In each village, a total of 100 children aged 9–12 years, 50 children aged 5–8 years, and 50 adults aged 20–55 years were randomly selected per survey to participate in parasitologic assessments. After obtaining written informed consent, participants were invited to provide a urine sample, produced between 10:00 and 14:00 hours. Urine samples were examined for macrohematuria, microhematuria, and presence of *S. haematobium* eggs by visual inspection, reagent strip testing (Haemastix; Siemens Healthcare Diagnostics GmbH, Camberley, Surrey, UK), and urine filtration, respectively.

**Figure 2.**
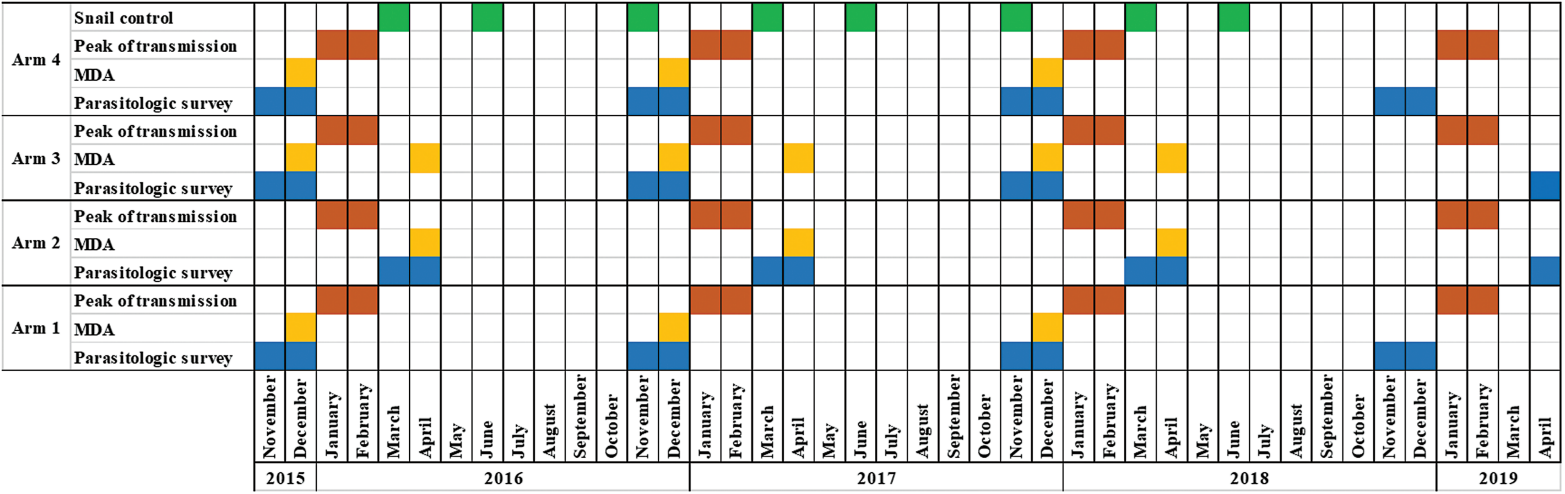
Timeline of study activities including parasitologic surveys (blue), MDA (yellow), peak of transmission (orange), and snail control surveys (green). Abbreviation: MDA, mass drug administration.

After the baseline survey conducted in all the study villages, follow-up surveys were done in villages of arms 1, 3, and 4 in November and December each year from 2016 to 2017. Villages of arm 2 were surveyed in March and April of each year from 2017 to 2018, according to the study design. The final parasitologic survey was carried out in November/December 2018 for arms 1 and 4, and April 2019 for arms 2 and 3, 1 year after the final MDA in each study arm (Figure 2).

### Snail Control Using Niclosamide

Snail control was implemented only in villages of arm 4 using niclosamide, the main molluscicide recommended by WHO [19, 20]. Malacologic surveys were conducted from 2016 to 2018, 3 times per year, in November, March, and June. Snails collected were identified by genus and, whenever possible, by species level, and densities were assessed at human-water contact sites. During each survey, those human-water contact sites found with *Bulinus* spp. (*B. truncatus* and *B. globosus*; the intermediate hosts of *S. haematobium* in this part of Côte d’Ivoire) were treated with niclosamide (concentration of 10 g/L) and were revisited the next day to determine whether snails might have survived.

### MDA Approaches

All individuals aged 5 years and above in the study villages were eligible for a free-of-charge preventive chemotherapy with a single 40 mg/kg oral dose of praziquantel, according to WHO guidelines. Both school-based treatment (SBT) and community-wide treatment (CWT) approaches were employed to increase the treatment coverage. Pupils were treated at school by trained teachers, whereas children not enrolled at school and adults were treated by trained community health workers who adopted a door-to-door approach.

The annual MDA in arms 1 and 4 and the first treatment in arm 3 occurred each December from 2015 to 2017. The second MDA in arm 3 and the annual treatment in arm 2 were each carried out in April from 2016 to 2018 (Figure 2). The treatment coverage per village was assessed by dividing the number of individuals aged 5 years and above who were treated by the total number of people of the relevant age group. We estimated the total population for each village based on the 2014 census, assuming an annual increase in population of 2.6% based on the estimated national growth rate. We assumed that 84% of the total population of each village would be in the age groups eligible for MDA based on the age distribution in the 2014 census.

### Statistical Analysis

Details on the number of clusters and participants and eligibility criteria have been published elsewhere [14]. In brief, data were double entered into an Excel spreadsheet (Microsoft Corporation; Redmond, Washington, USA), and cross-checked using EpiInfo version 3.4 (Centers for Disease Control and Prevention; Atlanta, Georgia, USA). The databases were uploaded and maintained on a central server (Open Data Kit) in Atlanta, Georgia, USA. For prevalence estimates, participants were considered infected if there was at least 1 *S. haematobium* egg discovered in 10 mL of filtered urine, examined under a microscope. The relative difference (% change) in *S. haematobium* prevalence between baseline and final surveys was calculated as follows: prevalence reduction rate = {[(prevalence at final survey − prevalence at baseline)/prevalence at baseline] × 100}. The arithmetic mean (AM) of infection intensity was estimated at village-level, expressed as *S. haematobium* eggs per 10 mL of urine. Infected children were classified as having light infection (1–49 eggs per 10 mL of urine) and heavy infection (≥50 eggs per 10 mL of urine), according to WHO thresholds [21]. *S. haematobium* egg counts were truncated at 1000 eggs per 10 mL urine. The reduction rate in infection intensity was calculated as follows: [(1 − AM eggs per 10 mL of urine at final survey/AM eggs per 10 mL of urine at baseline) × 100].

The primary analysis estimated differences between study arms in the final survey according to statistical analysis plans developed by SCORE investigators prior to data being available in this study [22, 23]. Differences in prevalence were evaluated using generalized estimating equations (GEE) for binary distributed outcomes with logit link and independent correlation structure to account for potential intra-class correlations within village clusters. Annual MDA before the peak of transmission (arm 1) was designated as the reference group. The secondary analysis used the same model but included baseline prevalence, sex, and age as additional covariates, and the model was weighted according to the number of observations in each village. Differences in egg counts were assessed using GEE for negative binomial distributed outcomes with log link and independent correlation structure. Adjusted and unadjusted models were estimated in the same way as the prevalence models. The primary analysis was done in R version 3.5.4.

We observed some imbalance among trial arms with respect to baseline prevalence. Hence, we decided to run, in addition to the prespecified models, an explorative analysis using inverse probability weighting (IPW) to adjust for baseline imbalances.

## RESULTS

### Study Flow

**Figure 3.**
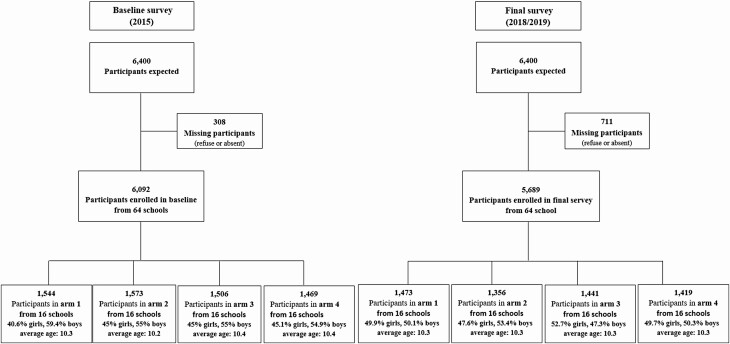
Study profile among children aged 9–12 years during baseline and last surveys. Final survey was carried out in November/December 2018 for arms 1 and 4 and in April/May 2019 for arms 2 and 3. Arm 1: Annual MDA with praziquantel before the peak schistosomiasis transmission season. Arm 2: Annual MDA with praziquantel after the peak schistosomiasis transmission season. Arm 3: MDA biannual treatment. Arm 4: Annual MDA with praziquantel before the peak schistosomiasis transmission season plus snails control with niclosamide. Abbreviation: MDA, mass drug administration.

The study, including the eligibility survey, was carried out between May 2015 and May 2019. Overall, 208 villages were subjected to an eligibility survey, of which 64 were selected for the study. No village dropped out during the study. At the parasitologic baseline survey, 6092 children aged 9–12 years were enrolled and 5689 at the final survey (Figure 3).

In addition, 3138 children aged 5–8 years were enrolled in the baseline and 3028 in the final survey. As regards adults aged 20–55 years, there were 3007 in the baseline and 2394 in the endline survey.

### Differences in Prevalence and Intensity of *S. haematobium* Infection

At the baseline survey, the prevalence of *S. haematobium* in children aged 9–12 years was 24.8% in arm 1, 10.1% in arm 2, 13.9% in arm 3, and 15.9% in arm 4. The AM egg counts per 10 mL of urine ranged from 5.7 eggs to 17.9 eggs between arms (Table 1). At the final survey, the prevalence and the AM egg count decreased in all study arms.

**Table 1.** Descriptive Results for Baseline and Final Survey per Study Arm (All Study Age Groups)

	Children Aged 9–12 Years				Children Aged 5–8 Years				Adults Aged 20–55 Years			
Variables	Arm 1	Arm 2	Arm 3	Arm 4	Arm 1	Arm 2	Arm 3	Arm 4	Arm 1	Arm 2	Arm 3	Arm 4
Number of villages	16	16	16	16	16	16	16	16	16	16	16	16
Number of people tested at baseline	1544	1573	1506	1469	820	788	804	726	727	761	762	759
Proportion female N (%) at baseline	627 (40.6)	708 (45.0)	672 (44.6)	662 (45.0)	427 (52.1)	383 (48.6)	378 (47.0)	342 (47.1)	434 (59.7)	473 (62.1)	461 (60.5)	443 (58.4)
Number of people infected at baseline	383	159	210	233	147	50	87	48	85	46	70	85
Prevalence at baseline (%)	24.8	10.1	13.9	15.9	17.9	6.4	10.8	6.6	11.7	6.0	9.2	11.0
Number of villages	16	16	16	16	16	16	16	16	16	16	16	16
Number of people tested in final survey	1473	1441	1419	1356	812	771	755	789	646	611	598	593
Proportion female N (%) in final survey	735 (49.9)	693 (48.1)	728 (51.3)	677 (49.9)	414 (51.0)	382 (49.5)	385 (51.0)	385 (48.8)	352 (54.5)	356 (58.3)	337 (56.3)	312 (52.6)
Number of people infected in final survey	111	50	8	46	66	27	19	22	11	14	11	3
Prevalence at final survey (%)	7.5	3.5	0.6	3.4	8.1	3.5	2.5	2.8	1.7	2.3	1.8	0.5
Absolute difference between prevalence at final survey and baseline	−17.3	−6.6	−13.4	−12.5	−9.8	−2.9	−8.3	−3.8	−10.0	−3.8	−7.3	−10.7
Relative difference in prevalence between final survey and baseline (% change)	−69.6	−65.7	−96.0	−78.6	−54.7	−44.9	−76.7	−57.8	−85.5	−62.1	−80.0	−95.5
Village-level arithmetic mean infection intensity at baseline (including zeros)	17.9	5.7	8.4	6.2	9.6	4.1	4.5	2.0	11.7	6.0	9.2	11.0
Village-level arithmetic mean infection intensity at final survey (including zeros)	11.7	1.6	0.3	0.7	8.2	3.2	0.5	0.6	0.4	0.3	0.3	0.1
Egg reduction rate (%)	34.6	71.9	96.4	88.7	14.6	22.0	88.9	70.0	96.6	95.0	96.7	99.1

Arm 1: Praziquantel annual mass drug administration (MDA) before peak of transmission. Arm 2: Praziquantel annual MDA after peak of transmission. Arm 3: Praziquantel MDA biannual treatment. Arm 4: Praziquantel annual MDA before peak of transmission plus snail control. N: case number.

At the final survey, the GEE model revealed that the difference in *S. haematobium* prevalence was significant between arms 1 and 3 (0.6% vs 7.5%; odds ratio [OR] = 0.07, 95% confidence interval [CI] = .02–.24). The observed prevalence in arms 2 and 4 were 3.5% and 3.4%, respectively, which were not statistically significantly different from arm 1. However, a significant difference was observed in egg counts between arm 1 and the other study arms. Adjusting for baseline imbalance with either covariate adjustment or IPW (Supplementary Appendix) did not change the interpretation of the results (Table 2).

**Table 2.** Differences in Prevalence and Intensity of *S*. *haematobium* Infection Between Study Arms at the Final Survey

		Prevalence		Intensity	
Age Group	Arms Compared	Unadjusted OR (95% CI)	Adjusted OR (95% CI)	Unadjusted CR (95% CI)	Adjusted CR (95% CI)
9- to 12-year-old	Arm 2 vs Arm 1	0.44 (.10–1.92)	1.67 (.41–6.88)	0.08 (.01–.66)	0.38 (.04–3.70)
	Arm 3 vs Arm 1	0.07 (.02–.24)	0.08 (.02–.33)	0.01 (.00–.08)	0.03 (.00–.25)
	Arm 4 vs Arm 1	0.43 (.11–1.74)	0.66 (.17–2.53)	0.04 (.01–.30)	0.1 (.01–.79)
5- to 8-year-old	Arm 2 vs Arm 1	0.41 (.08–2.13)	0.64 (.09–4.46)	0.25 (.02–2.68)	0.67 (.06–7.43)
	Arm 3 vs Arm 1	0.29 (.07–1.30)	0.34 (.08–1.56)	0.06 (.01–.41)	0.07 (.01–.51)
	Arm 4 vs Arm 1	0.23 (.04–1.23)	0.32 (.05–1.98)	0.03 (.00–.24)	0.03 (.00–.26)
20- to 55-year-old	Arm 2 vs Arm 1	1.35 (.43–4.29)	1.91 (.49–7.53)	0.73 (.18–3.01)	0.91 (.18–4.56)
	Arm 3 vs Arm 1	1.08 (.32–3.69)	1.27 (.37–4.41)	1.02 (.15–6.77)	0.77 (.10–5.86)
	Arm 4 vs Arm 1	0.29 (.08–1.02)	0.34 (.10–1.11)	0.37 (.04–3.18)	0.51 (.05–4.64)

The model was adjusted for baseline prevalence, sex, and age as additional covariates, and weighted according to the number of observations in each village. Arm 1: Praziquantel annual MDA before peak of transmission. Arm 2: Praziquantel annual MDA after peak of transmission. Arm 3: Praziquantel MDA biannual treatment. Arm 4: Praziquantel annual MDA before peak of transmission plus snail control.

Abbreviations: CI, confidence interval; CR, count ratio; MDA, mass drug administration; OR, odds ratio.

Among first-grade children and adults, no significant difference was observed in prevalence between study arms at the final survey (Table 2).

None of the study arms reached the goal of zero cases of *S. haematobium* infection (interruption of transmission) at the study end. However, the proportion of villages with zero cases of infection among children aged 9–12 years increased to >40% within all study arms between the baseline and the final surveys (Table 3).

**Table 3.** Proportion of Villages that Reached Incidence 0 New Cases of *S. haematobium* Infection and those that Reached Elimination as a Public Health Problem From Baseline to Final Survey per Study Arm (All the Study Age Groups Combined)

		Children Aged 9–12 Years		All Age Groups	
Study Year	Arm	Villages with Zero Cases N (%)	Villages EPHP N (%)	Villages with Zero Cases n (%)	Villages EPHP N (%)
Baseline	1	0 (0.0)	4 (25.0)	0 (0.0)	5 (31.3)
	2	2 (12.5)	10 (62.3)	0 (0.0)	10 (62.3)
	3	2 (12.5)	8 (50.0)	1 (6.3)	9 (56.3)
	4	2 (12.5)	7 (43.8)	0 (0.0)	6 (37.5)
Follow-up 1	1	5 (31.3)	12 (75.0)	3 (18.8)	12 (75.0)
	2	9 (56.3)	14 (87.5)	5 (31.3)	15 (93.8)
	3	10 (62.5)	16 (100)	6 (37.5)	16 (100)
	4	10 (62.5)	13 (81.3)	7 (43.8)	14 (87.5)
Follow-up 2	1	7 (43.3)	11 (68.8)	4 (25.0)	13 (81.3)
	2	10 (62.5)	15 (93.8)	7 (43.3)	15 (93.8)
	3	9 (56.3)	15 (93.8)	4 (25.0)	16 (100)
	4	9 (56.3)	12 (75.0)	5 (31.3)	14 (87.5)
Final survey	1	9 (56.3)	12 (75.0)	7 (43.3)	13 (81.3)
	2	7 (43.3)	11 (68.8)	4 (25.0)	13 (81.3)
	3	9 (56.3)	15 (93.8)	5 (31.3)	16 (100)
	4	8 (50.0)	14 (87.5)	7 (43.3)	14 (87.5)

Arm 1: Praziquantel annual MDA before peak of transmission. Arm 2: Praziquantel annual MDA after peak of transmission. Arm 3: Praziquantel MDA biannual treatment. Arm 4: Praziquantel annual MDA before peak of transmission plus snail control.

Abbreviations: EPHP, elimination as a public health problem (heavy infection <1%); MDA, mass drug administration; N, case number.

**Figure 4.**
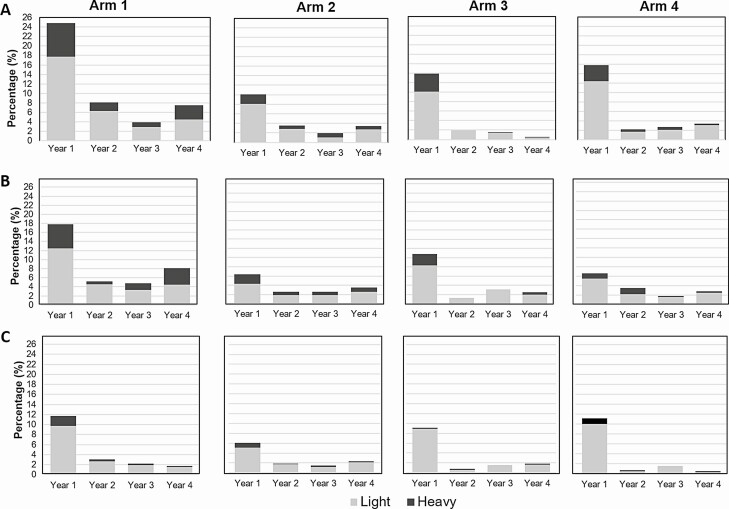
Overall prevalence stratified by infection intensity category by arm and by study year among (*A*) children aged 9–12 years, (*B*) children aged 5–8 years, and (*C*) adults aged 20–55 years. Total bar height represents *S. haematobium* infection prevalence in each year. Gray represents the prevalence of individuals with light intensity infections (1–49 eggs/10 mL of urine); and black represents prevalence of individuals with heavy intensity infections (≥50 eggs/10 mL of urine). Arm 1: Annual MDA with praziquantel before the peak schistosomiasis transmission season. Arm 2: Annual MDA with praziquantel after the peak schistosomiasis transmission season. Arm 3: MDA biannual treatment. Arm 4: Annual MDA with praziquantel before the peak schistosomiasis transmission season + snails control with niclosamide. Abbreviation: MDA, mass drug administration.

Heavy intensity infections decreased from baseline to final survey among all age groups in all arms (Figure 4). Proportion of villages with heavy infections <1% in final survey increased compared to baseline in all study arm (Table 3). However, 1 village alone recorded 36 of the 45 cases of heavy infection in arm 1 at the final survey.

### Snail Control With Niclosamide

The malacologic surveys made in June recorded the highest number of human-water contact sites visited per year. Both intermediate host species (ie, *B. truncatus* and *B. globosus*) were present, although *B. truncatus* was the predominant species. The percentage of human-water contact sites found with these snails varied between 7.4% and 33.3%, and all were treated with niclosamide during the months indicated (Table 4). The day after each treatment, the snails found in the human-water contact sites were all dead.

**Table 4.** Snail Control Coverage in the 16 Villages of Arm 4 Over Intervention Period With Niclosamide

Malacologic Survey Period	Human-Water Contacts sites	Human-Water Contact Sites with *B. *truncatus (%)	Human Water Contact Sites with *B. globosus* (%)	Total *Bulinus *spp.	Human-Water Contact Sites Treated with Niclosamide (%)
March 2016	23	5 (21.7)	0 (0)	837	5 (21.7)
June 2016	55	5 (9.1)	1 (1.8)	649	6 (10.9)
November 2016	47	6 (12.8)	3 (6.4)	329	9 (19.1)
March 2017	39	6 (15.4)	3 (7.7)	1201	9 (23.1)
June 2017	54	4 (7.4)	0 (0)	545	4 (7.4)
November 2017	41	6 (14.6)	4 (9.8)	1242	10 (24.4)
March 2018	27	6 (22.2)	3 (11.1)	754	9 (33.3)
June 2018	39	4 (10.2)	3 (7.7)	355	7 (17.9)

### MDA Coverage

MDA treatment coverage varied between study villages and from one treatment round to another. Table 5 shows MDA coverage by study arm over the course of the trial. The lowest coverage was observed in arm 4, varying from 65.6% in the first MDA round to 79.4% in the final round.

**Table 5.** Coverage of Mass Drug Administration (MDA) in Study Arm Over the 3-Year Intervention Period

Study Arm	Population	Treatment Period					
		December 2015	April 2016	December 2016	April 2017	December 2017	April 2018
1	Target population	17 226	…	17 657	…	18 099	…
	Treated population	13 930	…	15 168	…	18 347	…
	Coverage (%)	80.9		85.9		101.4	
2	Target population	…	21 381	…	21 916	…	22 464
	Treated population	…	16 391	…	18 325	…	14 401
	Coverage (%)		76.7		83.6		64.1
3	Target population	27 707	28 418	28 418	29 112	29 112	29 844
	Treated population	19 757	23 849	22 710	22 539	25 615	20 162
	Coverage (%)	71.3	83.9	79.9	77.4	88.0	67.6
4	Target population	27 031	…	27 707	…	28 399	…
	Treated population	17 718	…	18 757	…	22 543	…
	Coverage (%)	65.6		67.7		79.4	

## DISCUSSION

The WHO Strategic Plan 2012–2020 [6] called upon member states to attempt elimination of schistosomiasis as a public health problem and to interrupt transmission in selected areas by 2025. We implemented this cluster-randomized trial to assess 4 schedules to interrupt *S. haematobium* transmission in the northern and central parts of Côte d’Ivoire, characterized by seasonal transmission (transmission of schistosomiasis is not continuous throughout the year but rather linked to the season). In these settings, optimally timed drug administration and other interventions could increase the impact of schistosomiasis control and interruption of transmission seems possible. In line with WHO recommendations for interruption of transmission, MDA in our study was extended to all age groups. However, for the assessments, 9 to 12-year-old children were particularly targeted, as this age group is at high risk of schistosomiasis and sample collection is relatively straightforward.

MDA in schistosomiasis is advised during the dry season [24], but peak of transmission as a benchmark received little attention thus far. Our results showed that the timing of MDA (before or after peak transmission) had no effect on transmission. Previous studies concluded that the timing of MDA had a less pronounced effect than that of snails control [25]. We found a significant difference in *S. haematobium* prevalence at the final survey when comparing the arm with biannual treatment (arm 3) with the reference arm with annual MDA before peak transmission season (arm 1). A likely explanation of this observation is that more frequent treatment exerts pressure on interrupting transmission. Layering intermediate host snail control on top of annual MDA did not achieve a significant difference in reducing the prevalence and intensity of *S. haematobium* infection, as compared to the reference arm. This observation might be explained by the complexity of snail control, linked sometimes to the water surface size to be treated with niclosamide or to the difficulty to identify all human-water contact sites. In addition, niclosamide application is focal and sporadic, and hence, does not prevent the repopulation of treated areas by intermediate host snails. Our results corroborate findings obtained from a previous SCORE study in Zanzibar, Tanzania [26].

No study arm achieved the goal of zero cases of *S. haematobium* infection at the study end. Ongoing transmission in the study area might be explained by people who missed MDA, preschool-age children who have not been considered in treatments with praziquantel thus far, or in-migrating people infected with *S. haematobium*. It should be noted that some villages went from zero cases 1 year to new cases and even heavy infections the following year. These observations underscore the need to extend interventions for elimination to a longer time frame than in the current study and perhaps using additional control measures [27, 28]

Importantly, though, the secondary objective of eliminating *S. haematobium* as a public health problem was achieved in most of the study villages. However, there was a “persistent hotspot” [29, 30], where 36 cases of heavy intensity infections were clustered at the final survey. As already indicated by others, treatment alone fails to interrupt transmission in such contexts [31].

Beyond the limit of snail control articulated before, another limitation of our study is the underestimation of the true prevalence and infection intensity, because of the diagnostic approach used in the current trial. Indeed, there is considerable day-to-day variation of *S. haematobium* egg excretion and low-intensity infections are likely to be missed when only single urine samples are being processed in the laboratory [32, 33].

## CONCLUSION

All 4 intervention regimens investigated substantially reduced the prevalence and intensity of *S. haematobium* infection. The study arm with biannual MDA was the only approach tested that showed a significantly greater reduction in the prevalence and intensity of infection at the end of trial when compared to standard annual MDA scheduling. None of study interventions achieved the interruption of *S. haematobium* transmission. However, proportion of villages with zero cases of infection substantially increased at the study end within all study arms and most of them reached the goal of *S. haematobium* elimination as a public health problem. Snail control did not significantly improve the effect of MDA in our study. However, we recommend assessment of snail control combined with a more intensive MDA program for a longer period to better appreciate a potential impact on transmission interruption.

## Supplementary Data

Supplementary materials are available at *Clinical Infectious Diseases* online. Consisting of data provided by the authors to benefit the reader, the posted materials are not copyedited and are the sole responsibility of the authors, so questions or comments should be addressed to the corresponding author.

ciab787_suppl_Supplementary_Appendix

## Notes

***Acknowledgments.*** The authors thank the SCORE secretariat (Daniel G. Colley, Carl H. Campbell Jr, Charles H. King, and Sue Binder), and Abdoulaye Meïté and his team from the “Programme National de Lutte contre les Maladies Tropicales Négligées à Chimiothérapie Préventive” in Côte d’Ivoire. They thank the laboratory technicians who contributed to this work and all the teachers and village leaders in the project areas for their contribution to population sensitization.

***Authors’ contributions.*** M. O., J. H., J. T. C., J. U., and E. K. N. designed the study protocol according to SCORE guidelines. M. O., F. K. B., N. R. D., Y.-N. T. T.-B., C. K. K., R. K. A., N. K., and N. G.-C. collected data. J. H. and P. K. Y. assisted with data management and statistical analysis. M. O. wrote the first draft of the manuscript. All authors contributed to the manuscript revision and approved the final version prior to submission.

***Financial support.*** This study was supported by the Schistosomiasis Consortium for Operational Research and Evaluation (SCORE) through a grant provided to the University of Georgia Research Foundation, Inc. from the Bill & Melinda Gates Foundation (grant numbers: prime award number 50816; subaward number RR374-053/4787986). Drugs were provided by the Schistosomiasis Control Initiative (SCI) based at Imperial College London, UK.

***Potential conflicts of interest.*** The authors: No reported conflicts of interest. All authors have submitted the ICMJE Form for Disclosure of Potential Conflicts of Interest. Conflicts that the editors consider relevant to the content of the manuscript have been disclosed.
