# Supplementary material for: Effectiveness of Four Different Interventions Against Schistosoma haematobium in a Seasonal Transmission Setting of Côte d’Ivoire: A Cluster Randomized Trial
Source: Clin Infect Dis. 2021 Sep 14;74(12):2181–90. doi: 10.1093/cid/ciab787 (PMC9258925; doi:10.1093/cid/ciab787)

Exploratory analysis – Inverse probability weighted (IPW) analysis

Odds ratios and corresponding confidence intervals for the unadjusted (black), covariate adjusted (orange) and IPW (red) analysis to accunt for imbalance in baseline prevalences. Black numbers show the observed prevalences in each treatment arm and red numbers show the prevalences in each arm after IPW.
The weighted prevalences at baseline (red numbers, upper panel within each age category) indicate, that the imbalance was largely removed after IPW. The only exception is the comparison 'Arm 1 vs 4' in the age category 5-8yrs, where considerable imbalance remained after IPW. All analyses were adjusted for clustering using GEE models.

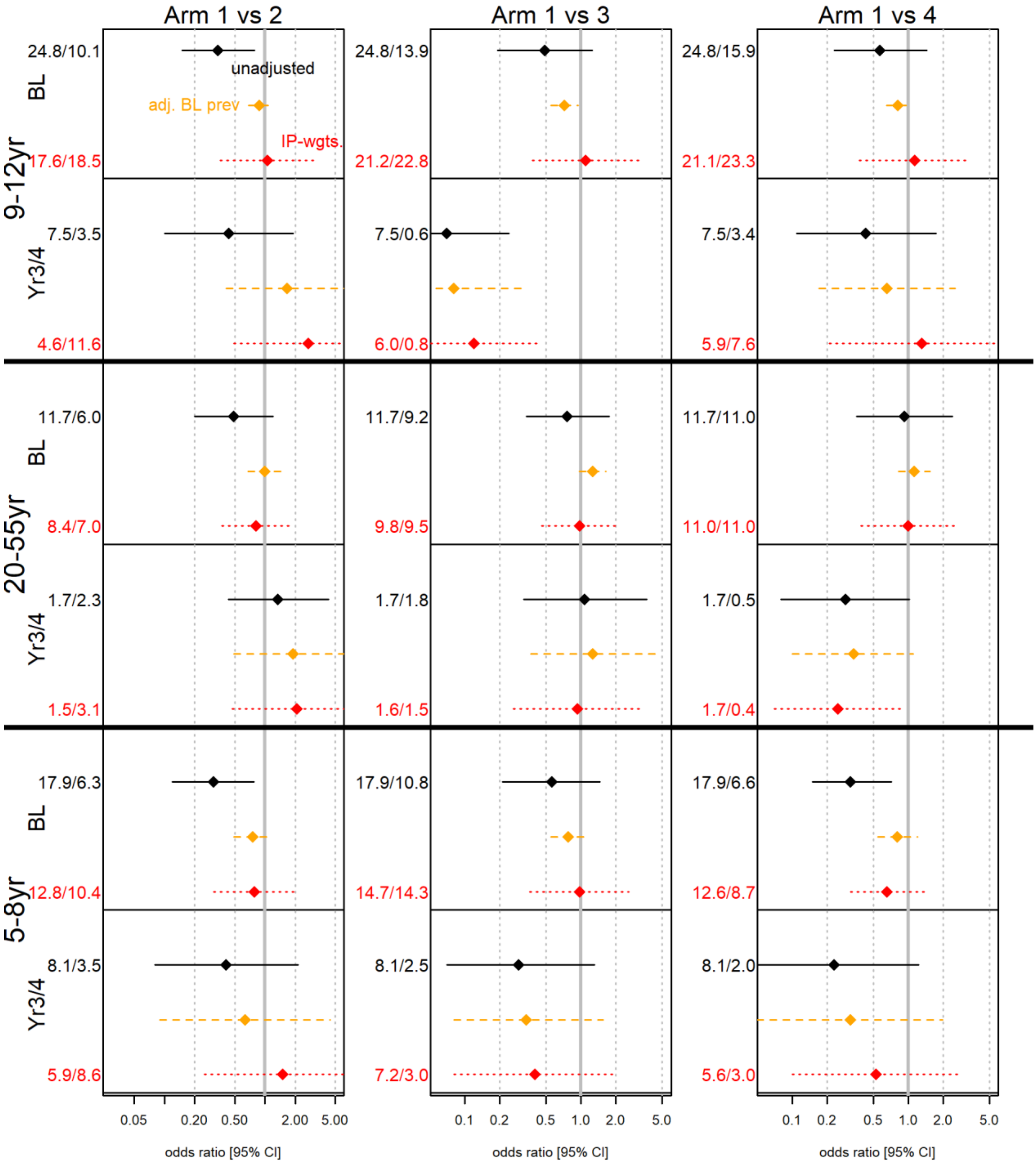

Supplement: ciab787_suppl_Supplementary_Appendix [file ciab787_suppl_supplementary_appendix.pdf]
